# Seasonality of sand flies (Diptera: Psychodidae) and *Leishmania* DNA detection in vector species in an area with endemic visceral leishmaniasis

**DOI:** 10.1590/0074-02760160438

**Published:** 2017-04

**Authors:** Lara Saraiva, Camila Gonçalves Leite, Ana Cristina Vianna Mariano da Rocha Lima, Luiz Otávio Alves de Carvalho, Agnes Antônia Sampaio Pereira, Jerônimo Marteleto Nunes Rugani, Felipe Dutra Rego, Célia Maria Ferreira Gontijo, José Dilermando Andrade

**Affiliations:** 1 Fundação Oswaldo Cruz-Fiocruz, Centro de Pesquisas René Rachou, Grupo de Estudos em Leishmanioses, Belo Horizonte, MG, Brasil; 2 Prefeitura Municipal de Belo Horizonte, Gerência de Controle de Zoonoses, Belo Horizonte, MG, Brasil

**Keywords:** visceral leishmaniasis, Lutzomyia longipalpis, Belo Horizonte

## Abstract

**BACKGROUND:**

Leishmaniases are a serious health problem in southeast Brazil, including the city of Belo Horizonte (BH), Minas Gerais state (MG), where there are high rates of incidence and mortality due to visceral leishmaniases. BH is divided into nine sanitary districts (SD) of which one, the Venda Nova SD, was selected for this study because it has high rates of positivity for canine leishmaniasis and high incidence of human leishmaniasis.

**OBJECTIVES:**

This study aimed to survey the sand fly fauna in Venda Nova SD from August 2011 to July 2013 and perform a descriptive analysis of the vector population.

**METHODS:**

The sampling was carried out using automatic HP light traps at all covered areas of the Venda Nova SD, in a total of eighteen light traps. Sampled specimens were identified following Galati (2003), and females were submitted to molecular techniques for the detection and identification of *Leishmania* DNA. A simple environmental description was done for it area and Kernel estimation was used to infer vector density for each study site.

**FINDINGS:**

A total of 2,427 sand fly specimens belonging to eight species and five genera were collected of which 95.3% were *Lutzomyia longipalpis.* The seasonal variation curve was delineated by this species. *Lu. longipalpis* was the most abundant at all collection points and in all months of the study, and exhibited a natural infection rate of 1.01% for *Leishmania infantum* and 1.77% for *Leishmania braziliensis*.

**MAIN CONCLUSIONS:**

The results show the presence and adaptation of *Lu*. *longipalpis* to the anthropic environment of BH and reinforces its role as the main vector of *L. infantum* in the region.

The principal etiological agent of visceral leishmaniases (VL) in the Americas is *Leishmania* (*Leishmania*) *infantum* (Nicolle, 1908) and the main vector of this agent is the sand fly *Lutzomyia* (*Lutzomyia*) *longipalpis* (Lutz & Neiva, 1912). Distinctively, American cutaneous leishmaniasis (ACL) features a range of etiological agents and sand fly vectors. The clinical presentations, prognoses, epidemiological patterns, and cycles of occurrence vary with the species of parasites involved. Thus, being a multifactorial phenomenon, the species of parasite greatly determines the clinical features of ACL (WHO 2010).

VL has considerable epidemiological importance in Brazil, with an annual rate of incidence between 4,200 to 6,300 cases yearly. The incidence of ACL is much higher ranging from 72,800 to 119,600 cases annually. It should be noted, however, that these incidence are surely underestimates because of the problems that exist with underreporting by the public health system in Brazil (Alvar et al. 2012) Furthermore, the areas of epidemiological and public health significance for these diseases are spreading, including urban areas in Brazil. In the southeast of Brazil, the spread of leishmaniasis has been accompanied by a significant increase in the number of cases. The municipality of Belo Horizonte (BH), Minas Gerais (MG) state, and its metropolitan region are one of the most serious examples of the spread of leishmaniasis in southeast Brazil. The first autochthonous VL cases recorded in the city of BH were in 1994. During the subsequent 19 years of the occurrence of the disease the municipality has amassed 1,526 reported cases, with an average annual death rate of 20% during the past five years (PBH 2014).

The municipality of BH is divided into nine sanitary districts (SD), each with their own socio-economical, epidemiological and environmental characteristics. Each SD displays their own epidemiological pattern of the number of human VL cases, rate of canine positivity, and composition and seasonal variation of sand fly populations (de Souza et al. 2004, Resende at al. 2006, Saraiva et al. 2011, PBH 2014). Therefore, detailed studies on the sand fly fauna and the epidemiological characteristics of VL in each SD might, together, lead to a better understanding of the occurrence of this disease. Each SD is divided into coverage areas based on the population assisted at each of the health centres. Studies performed in the metropolitan region of BH have already documented the sand fly vectors of *L.* (*L.*) *infantum* and *Leishmania* (*Viannia*) *braziliensis* (Vianna, 1911) (Carvalho et al. 2010).

Venda Nova SD, selected for this study, has high rates of positivity for canine leishmaniasis and high incidence of human leishmaniasis. The last sand fly survey carried out in Venda Nova SD occurred from 2001-2003, and utilised two traps at each of three sampling points in only three coverage areas. The survey found *Lu. longipalpis* to be the most predominant species, but *Nyssomyia intermedia* (Lutz & Neiva, 1912), *Nyssomyia whitmani* (Antunes & Coutinho, 1939), *Lutzomyia firmatoi* (Barretto, Martins & Pellegrino, 1956) and *Evandromyia sallesi* (Galvão & Coutinho, 1939) were also reported, although less abundant (de Souza et al. 2004).

The present study aimed to perform a more detailed survey of sand flies in Venda Nova SD by including all coverage areas and using a simple descriptive methodology but with analysis at the global level, which could be used in the health service of zoonoses control. The detailed knowledge of the seasonal fluctuation curves of the vector species of *Leishmania*, as well as the knowledge of the areas of greatest occurrence of vector abundance can contribute to the control actions of leishmaniosis control in urban areas. Since the chronic pattern of infection development makes the occurrence of cases an indicator difficult to be used alone.

In this work 18 light traps were used in a single SD of the municipality of BH, in monthly collections of three consecutive days during two years, in the previous studies the quantitative of traps was relatively smaller, as well as the temporal sample effort. De Souza et al. (2004) used 54 traps throughout the municipality with collections of one day, for two years. Resende et al. (2006) used nine traps in three SDs with monthly collections of one day and Saraiva et al. (2011) used 24 traps in only one SD with monthly collections of one day during a sample year.

## MATERIALS AND METHODS

**Fig. 1:**
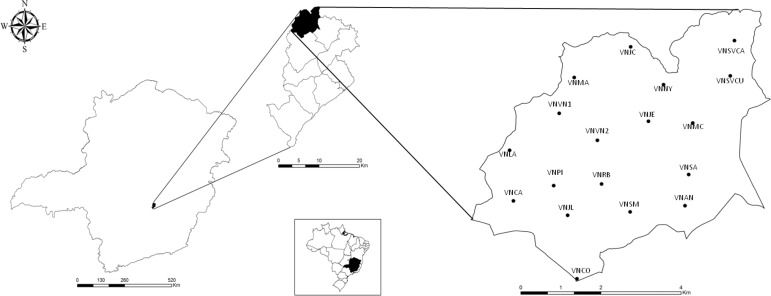
: map showing the location of the city of Belo Horizonte, the Venda Nova Sanitary District, and the local collection points in each coverage area.

*Study area* - The Venda Nova SD corresponds to northern portion of the municipality of BH and was the earliest region of BH to be developed. It is divided into 16 areas of coverage based on the population assisted at each of the health centres: Andradas (VNAN); Céu Azul (VNCA), Copacabana (VNCO), Jardim dos Comerciários (VNJC), Jardim Europa (VNJE), Jardim Leblon (VNJL), Lagoa (VNLA), Mantiqueira (VNMA), Minas Caixa (VNMC), Nova Yorque (VNNY), Piratininga (VNPI), Rio Branco (VNRB), Santo Antônio (VNSA), Santa Mônica (VNSM), Serra Verde (VNSVCU and VNSVCA) and Venda Nova (VNVN1 and VNVN2) (PBH 2014) (Fig. 1).

*Sand fly collection* - Procedures for collecting sand flies were approved by the Ministério do Meio Ambiente do Brasil (Ministry of Environment of Brazil) - (SISBIO license number 15,237). For the analysis of sand fly populations, 18 light traps were arranged uniformly within the SD; one trap was centrally located in each health centre coverage area. In two sites, Serra Verde (VNSV) and Venda Nova (VNVN), two traps were used because the geographical areas of these sites did not enable the location of a single central point. A simple environmental characterisation was performed at each collection point by recording environmental characteristics related to phlebotomine occurrence at a locality. These characteristics included the presence of vegetation and the presence of water, both of which relate to breeding sites and the supportive environment for phlebotomine. The presence of livestock, the presence of a hen house, and the presence of a dog kennel are all related to providing a source of food for females. The traps when possible were placed inside the shelters of the animals, or as close as possible to the environments of interest, a few metres away, at most about 10 metres. The environmental characteristics of the collection point are briefly described in (Table I).

**TABLE I:** Environmental characteristics of the collection localities for collection of phlebotomine flies in Venda Nova Sanitary District, Belo Horizonte, from August 2011 to July 2013

Coverage área	Location	Sites considered favorable for the collection of phlebotomine sand flies			

		Presence of vegetation	Presence of hen house	Presence of dog kennel	Presence of stream, pond or dam
VNAN	Backyard	Yes	No	No	No
VNCA	Backyard	Yes	No	Yes	No
VNCO	Backyard	Yes	Yes	Yes	Yes
VNJC	Backyard	Yes	Yes	Yes	No
VNJE	Backyard	No	No	Yes	No
VNJL	Backyard	Yes	No	Yes	Yes
VNLA	Health centre backyard	Yes	No	No	No
VNMA	Backyard	Yes	Yes	Yes	No
VNMC	Backyard	Yes	No	Yes	No
VNNY	Backyard	Yes	No	Yes	Yes
VNPI	Health centre backyard	Yes	No	No	No
VNRB	Backyard	No	No	Yes	No
VNSM	Farmstead	Yes	Yes	Yes	Yes
VNSA	Farmstead	Yes	Yes	Yes	No
VNSVCA	Plant nursery	Yes	No	No	Yes
VNSVCU	Backyard	Yes	Yes	Yes	Yes
VNVN1	Backyard	Yes	Yes	Yes	No
VNVN2	Farmstead	Yes	Yes	Yes	Yes

VNAN: Venda Nova Andradas; VNCA: Venda Nova Céu Azul; VNCO: Venda Nova Copacabana; VNJC: Venda Nova Jardim dos Comerciários; VNJE: Jardim Europa; VNJL: Venda Nova Jardim Leblon; VNLA: Venda Nova Lagoa; VNMA: Venda Nova Mantiqueira; VNMC: Venda Nova Minas Caixa; VNNY: Venda Nova Nova Yorque; VNPI: Venda Nova Piratininga; VNRB: Venda Nova Rio Branco; VNSA: Venda Nova Santo Antônio; VNSM: Venda Nova Santa Mônica; VNSVCU/VNSVCA: Venda Nova Serra Verde; VNVN1/VNVN2: Venda Nova.

Traps were randomly distributed, thus allowing the assessment of locations as either favorable or not for the development and collection of sand flies. The principle aim trapping was the uniformity of the sample effort in geographical area (Table I).

Sampling was carried out by endemic disease control agents and were integrated with the regular activities of Zoonoses Control in Venda Nova SD. The sampling period was from August 2011 to July 2013, with a total sampling effort of 31,104 hours. Phlebotomine collections were performed with automatic HP traps (Pugedo et al. 2005) in 12 h nocturnal collections of three consecutive days, performed monthly for two years. In fixed weeks, always in the third week of each month. The classification proposed by Galati (2003) was used to identify the specimens. Females of *Ev. sallesi* and *Ev. cortelezzii* (Brethes, 1923) were recorded as belonging to the “cortelezzii complex” when morphological differentiation was inconclusive.

*Detection of Leishmania DNA in trapped females* - Trapped females were stored in DMSO 6% until DNA extraction using the Puregene® QIAGEN Gentra kit. All samples were analysed individually and Polymerase chain reaction (PCR) for the molecular target internal transcribed spacer I (ITS-1) (Schönian et al. 2003) and the small subunit (SSU) (Cruz et al. 2002) were performed following the methodology modified by (Rego et al. 2014).

The positive controls were DNA of four reference strains: *L. braziliensis* (MHOM/BR/75/M2903), *L. guyanensis* (MHOM/BR/75/M4147), *L. infantum* (MHOM/BR/74/PP75), and *L. amazonensis* (IFLA/BR/67/PH8) at a concentration of 20 ng/mL. For species identification the amplified products of the ITS-1 reactions were digested by the enzyme Hae III as described by Schöniam et al. (2003).

If the restriction fragment analysis did not produce visible DNA bands, the whole fragment resulting from the PCR amplification of the ITS-1 or SSU region was sequenced. To purify the product QIAquick® PCR Purification Kit (Qiagen) was used following the manufacturer’s instructions. Next, a mixture was prepared containing 1 μL of purified products, 1 μL of each primer at a concentration of 5 pmol, antisense or sense (in separate tubes), 1 μL 5x Sequencing Buffer, 1 μL of BigDye® Terminator v3.1 Cycle Sequencing, and distilled water for a final volume of 10 μL. The program used 35 cycles alternating between 95ºC for 15 s and 65ºC for 15 s. The ITS-1 fragment sequences were read using an ABI 3730xl DNA Analyser automatic DNA sequencer.

*Analyses* - All data were analysed descriptively using Microsft Excel software and statistical analyses were performed using the program Graph Pad Prism. The ecological indices of richness, diversity and equitability were calculated using PAST software.

The entomological sampling sites were georeferenced using GPS - GARMIN eTrex-H for spatial analysis of population density of suspected and confirmed vector species. Coordinates and presence/absence data of vectors in each site were analysed using ArcGIS 9.3 software.

Kernel estimation was used to infer vector density for each study site. This is a nonparametric statistical method of interpolation that identifies sites with the highest occurrence of a given event (MS 2007). In the present study, sand fly species were categorised as vector (suspected or confirmed) and non-vector species *Leishmania* sp.

Climatological data were obtained from the Ministério da Agricultura e Abastecimento, Instituto Nacional de Meteorologia (Ministry of Agriculture and Supply, National Institute of Meteorology).

## RESULTS

*Sand fly fauna* - A total of 2,427 sand flies of eight species belong to five genera were collected. The ratio of males to females was 4.26:1. The species with the highest abundance was *Lu. longipalpis* (2,313), representing 95.30% of the specimens collected, followed by *Ev. cortelezzii* (1.52%), *Ev. sallessi* (0.66%), and *Ny. whitmani* (0.37%) (Table II).

**TABLE II:** Total number of sand flies collected by coverage area and sex in Venda Nova Sanitary District, Belo Horizonte, from August 2011 to July 2013

Sand fly species	Study localities (Coverage areas)																		Sex		Total
	
	VNAN	VNCA	VNCO	VNJC	VNJE	VNJL	VNLA	VNMA	VNMC	VNNY	VNPI	VNRB	VNSM	VNSA	VNSVCA	VNSVCU	VNVN1	VNVN2	♂	♀	
*cortelezzii* complex	1	0	0	0	0	0	1	0	2	0	1	0	2	6	0	1	4	1	-	19 (0.78)	19 (0.78)
*Evandromyia cortelezzii*	1	1	1	3	5	1	0	3	4	0	1	0	5	5	0	0	4	3	28 (1.15)	9 (0.37)	37 (1.52)
*Ev. lenti*	0	0	0	0	0	0	0	0	0	0	0	0	1	0	0	1	0	0	-	2 (0.08)	2 (0.08)
*Ev. sallessi*	1	0	1	0	2	0	0	0	3	1	0	0	5	0	0	0	3	0	4 (0.16)	12 (0.49)	16 (0.66)
*Evandromyia* sp.	0	0	0	0	15	0	0	0	0	0	0	0	0	0	0	0	0	0	-	15 (0.62)	15 (0.62)
*Lutzomyia longipalpis*	9	17	45	29	213	5	1	28	111	18	3	11	1229	507	0	9	58	20	1919 (79.07)	394 (16.23)	2313 (95.30)
*Lu. cavernicola*	0	0	0	0	0	0	0	0	0	0	0	0	1	0	0	0	0	0	-	1 (0.04)	1 (0.04)
*Lutzomyia sp.*	0	0	0	2	3	0	0	0	0	0	0	0	6	1	0	0	1	0	8 (0.33)	5 (0.21)	13 (0.54)
*Micropygomyia schreiberi*	0	0	0	0	0	0	0	0	0	0	0	0	0	0	0	1	0	0	1 (0.04)	-	1 (0.04)
*Nyssomyia whitmani*	0	0	0	4	0	0	0	0	0	0	1	0	3	0	0	1	0	0	6 (0.25)	3 (0.12)	9 (0.37)
*Sciopemyia sordellii*	0	0	0	0	0	0	0	1	0	0	0	0	0	0	0	0	0	0	-	1 (0.04)	1 (0.04)
Total	12	18	47	38	238	6	2	32	120	19	6	11	1252	519	0	13	70	24	1966 (81.01)	461 (18.99)	2427 (100.00)

VNAN: Venda Nova Andradas; VNCA: Venda Nova Céu Azul; VNCO: Venda Nova Copacabana; VNJC: Venda Nova Jardim dos Comerciários; VNJE: Jardim Europa; VNJL: Venda Nova Jardim Leblon; VNLA: Venda Nova Lagoa; VNMA: Venda Nova Mantiqueira; VNMC: Venda Nova Minas Caixa; VNNY: Venda Nova Nova Yorque; VNPI: Venda Nova Piratininga; VNRB: Venda Nova Rio Branco; VNSA: Venda Nova Santo Antônio; VNSM: Venda Nova Santa Mônica; VNSVCU/VNSVCA: Venda Nova Serra Verde; VNVN1/VNVN2: Venda Nova.

**Fig. 2:**
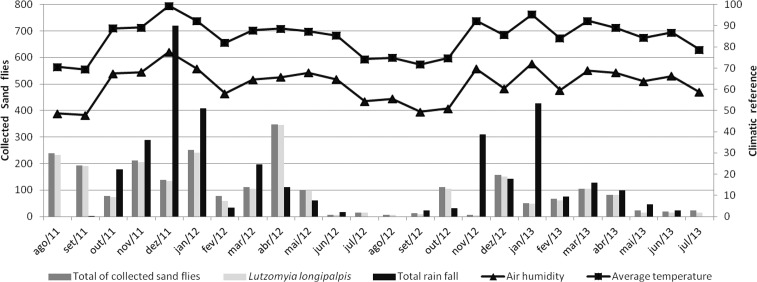
: seasonal variation of sand flies and climate parameters of total precipitation, relative humidity and mean temperature in Venda Nova Sanitary District from August 2011 to July 2013.

Differences among the coverage areas in the abundance of specimens collected were due to the number of *Lu. longipalpis* specimens. One location, VNSVCA, had no phlebotomine specimens collected, whereas all the other locations had at least two individuals of two species collected. In the first year of sampling, the greatest numbers of sand flies were collected during the months of August, January and April, whereas the cold and dry months had the lowest numbers. The second year had lower abundances of collected sandflies as a general pattern. The months with the greatest numbers of sand flies collected were October, December and March. Analyses of the seasonal curve with climatic parameters showed that peaks of sand fly abundance coincided with, or followed, periods of high rainfall; a similar pattern was observed for relative humidity. Higher temperatures also coincided with peaks of sand fly occurrence (Fig. 2). However, in spite of these apparent correlations, none of the climatic parameters showed a statistically significant correlation with the seasonal curve of sand flies in Venda Nova SD [Temperature - (p = 0.1052 - Pearson correlation test), Precipitation - (p = 0.3798 - Spearman correlation test), Air relative humidity (p = 0.8506 - Pearson correlation test)].

The seasonal pattern of occurrence of *Lu. longipalpis* varied remarkably between years and among the coverage areas. In the first year of sampling (August 2011 to July 2012), *Lu. longipalpis* had the highest abundance and VNSM was the locality with the highest relative abundance with 71.53% of the specimens collected. The total collection curve for this time period was largely determined by specimens collected from VNSM and VNJE, which accounted for 11.53% of the total specimens collected. In the second year of sampling VNSA was the coverage area with the highest percentage of collected specimens at 68.84%, while VNSM accounted for only 2.12% (Table II, Fig. 2).

The species accumulation curve analysis for Venda Nova SD reached saturation at the 19th sampling event with only eight species. These data, combined with the seasonal pattern, demonstrate the dominance of *Lu. longipalpis* in the urban environment. In Venda Nova SD, the species seasonal variation curve was determined by *Lu. longipalpis* (Fig. 2, Table III).

**TABLE III:** Margalef diversity and equitability J indices by sampling event in Venda Nova Sanitary District, from August 2011 to July 2013

Sampling	First year											

	1º	2º	3º	4º	5º	6º	7º	8º	9º	10º	11º	12º
Richness	2	3	3	3	4	4	3	3	2	2	1	1
Species accumulation	2	3	4	5	5	5	5	6	6	6	6	6
Margalef diversity	0.18	0.38	0.46	0.37	0.61	0.55	0.48	0.43	0.17	0.22	0.00	0.00
Equitability_J	0.15	0.08	0.22	0.14	0.12	0.09	0.25	0.13	0.03	0.08	0.00	0.00

Sampling	Second year											

	13º	14º	15º	16º	17º	18º	19º	20º	21º	22º	23º	24º

Richness	1	3	2	1	2	1	3	1	1	3	2	3
Species accumulation	6	7	7	7	7	7	8	8	8	8	8	8
Margalef diversity	0.00	0.87	0.21	0.00	0.20	0.00	0.48	0.00	0.00	0.63	0.36	0.68
Equitability_J	0.00	0.58	0.30	0.00	0.17	0.00	0.24	0.00	0.00	0.72	0.34	0.64

Margalef diversity and Equitability J indices adequately described the sand fly communities in Venda Nova SD. Low species diversity and the marked dominance of *Lu. longipalpis* are reflected in the low values for these indices over the sampling period (Table III).

The distribution of sand flies in the urban environment is unequal with respect to the abundance of specimens collected; a greater number of specimens were collected in areas with more attractive environmental characteristics, as was the case with VNSM. However, there are some locations with environmental characteristics considered highly attractive but had a low percentage of the total specimens collected, as was the case with VNSVCU. *Lu. longipalpis* was collected in 15 of the 16 study localities in Venda Nova SD and was the most abundant species in these 15 locations (Tables I-II).

**Fig. 3:**
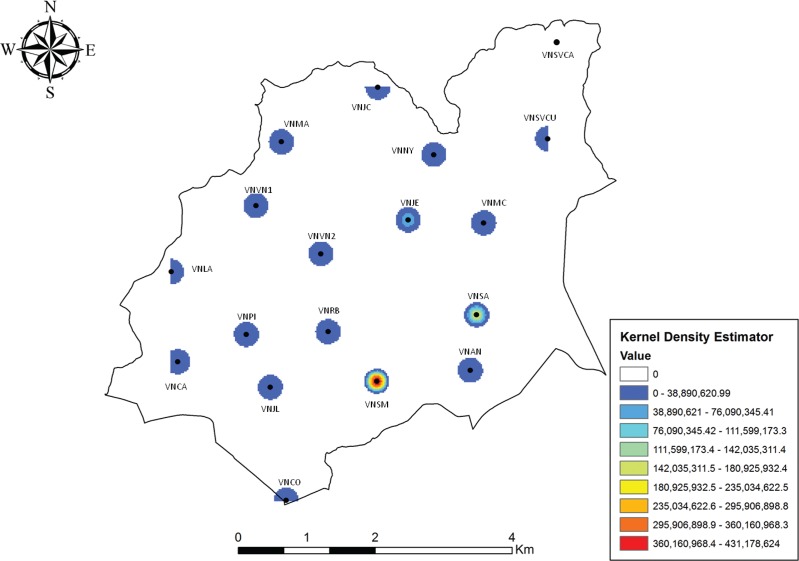
: map showing the Venda Nova Sanitary District and the Kernel density analysis of vectors.

The distribution maps of vector and probable-vector species indicate a concerning epidemiological situation in all coverage areas. Throughout Venda Nova SD and three specific coverage areas (VNSM, VNSA and VNJE) *Lu. longipalpis* had a high spatial accumulation. In this analysis, the map of vector occurrence can be used to evaluate the map of occurrence of *Lu. longipalpis* given the high abundance of this species in the SD (Fig. 3).

*Leishmania DNA detection in female sand flies* - Of the 461 females collected in the Venda Nova SD, 12 had positive amplifications for intergenic region ITS-1. Two samples were also submitted to the SSU-RNA target reaction and had positive results. All positive samples were of *Lu. longipalpis*. Four of the samples identified the presence of *L. infantum* DNA, seven identified *L. braziliensis* DNA, and one sample could only be characterised as belonging to the family Tripanossomatidae, this sample was positive only for ITS-1 reaction (Table IV).

**TABLE IV:** *Leishmania* DNA detection in female sand flies collected in Venda Nova Sanitary District, from August 2011 to July 2013

Sand flie species	Area	PCR-ITS1	RFLP - ITS1	ITS1 Sequencing	Nested PCR -SSU RNA	SSU-RNA sequencing
*Lutzomyia longipalpis*	VNJE	positive	*Leishmania braziliensis*	low quality	not performed	not performed
*Lu. longipalpis*	VNJE	positive	undefined profile	low quality	not performed	not performed
*Lu. longipalpis*	VNJE	positive	*L. braziliensis*	low quality	not performed	not performed
*Lu. longipalpis*	VNJE	positive	*L. braziliensis*	low quality	not performed	not performed
*Lu. longipalpis*	VNJE	positive	undefined profile	*L. infantum* (ID: 94%, Ref: KC998879.1)	not performed	not performed
*Lu. longipalpis*	VNSM	positive	undefined profile	not performed	positive	Complexo donovani - *L. infantum* (ID: 99%, Ref: EU825208.1)
*Lu. longipalpis*	VNSM	positive	*L. infantum*	*L. Infantum* (ID: 100%, Ref: KC347301.1)	positive	low quality
*Lu. longipalpis*	VNSM	positive	*L. braziliensis*	*Leishmania* sp. (ID: 91%, Ref: HQ830349.1)	not performed	not performed
*Lu. longipalpis*	VNSM	positive	not performed	*L. Infantum* (ID:88%, Ref: KC998879.1)	not performed	not performed
*Lu. longipalpis*	VNMC	positive	*L. braziliensis*	*L. braziliensis* (ID: 85%, Ref: FN398338.1)	not performed	not performed
*Lu. longipalpis*	VNMC	positive	*L. braziliensis*	*Leishmania* sp. (ID: 93%, Ref: FJ753382.1 )	not performed	not performed
*Lu. longipalpis*	VNMC	positive	*L. braziliensis*	low quality	not performed	not performed

ITS1: internal transcribed spacer I; PCR: polymerase chain reaction; RFLP: restriction fragment length polymorphism; SSU: small subunit; VNJE: Venda Nova Jardim Europa; VNMC: Venda Nova Minas Caixa; VNSM: Venda Nova Santa Mônica.

Just three coverage areas, VNSM, VNMC and VNJE, had specimens that were positive for *Leishmania* DNA. VNMC had three positive specimens and VNSM had four positive specimens and VNJE had five. In VNMC only *L. braziliensis* was identified, in VNSM and VNJE both *L. braziliensis* and *L. infantum* were found. The total natural infection rate in the SD was 2.38%. The overall infection rate for *Lu. longipalpis* was 2.79%, with *L. infantum* and *L. braziliensis* having infection rates of 1.01% and 1.77%, respectively.

## DISCUSSION

Considering how long humans have been on the planet, urbanisation is a very recent phenomenon and understanding its effects on human health remains a major challenge for public health. This perspective becomes even more dramatic when one considers urbanisation in underdeveloped countries in the tropics (Lau et al. 2010).

The current epidemiological importance of VL in large Brazilian cities illustrates this challenge. Despite the great number of studies on various aspects of this disease and the implementation of control measures, human and canine infections remain a serious problem (MS 2006).

In addition, performing reliable evaluations of the impact of control measures in epidemiological scenarios where the disease is occurring remains problematic. A number of factors may influence the quality of control measures but there is no standardized procedure to evaluate the quality of the actions that are implemented (MS 2006). Complex statistical models already used for epidemiological description of leishmaniasis are beyond the objectives of this study (Araújo et al. 2013). The study was developed with a methodology that could be reproduced in the field of health services in Brazilian municipalities.

Venda Nova SD is located in the northern portion of the city of BH and is a mosaic of urbanisation, slums, urban agglomerations, and areas with markedly rural characteristics. In this study, the simple environmental characterisation that was performed demonstrates that urban organisation might create environments that favor the occurrence of certain synanthropic species involved in disease cycles that represent risks to human health.

From the 18 sand-fly collection localities, eight had hen houses, three had other animals such as cattle, horses and pigs, and fourteen had dogs. Inadequate management of hen houses can favor the presence of animals that could represent a risk to human, such as phlebotomines and rodents (Lainson & Rangel 2005). The domestic dog is recognised as the main urban reservoir of *L. infatum* in Brazil, and their role as a reservoir for *L. brazileinzis* needs to be clarified (Marzochi & Marzochi 2004).

Breeding certain animals, such as pigs, cattle and horses, in urban areas is not prohibited by the health code of the city of BH, however, such species bred inappropriately might favor the occurrence of an unhealthy environment that favors the development of urban health problems. Thus, according to the health code, it is expected that citizens act responsibly with their property and should prevent conditions that favor the occurrence of synanthropic animals and other environmental conditions that can represent a health risk to the community (PBH 2014). For example, in the three places where there were livestock animals breeding, there was an accumulation of feces, for which there was inadequate treatment facilities.

Only two of the collection sites had no vegetation present, but seven collection sites were close to bodies of water. Phlebotomines typically are associated with locations possessing vegetation, decomposing organic matter, moisture and shaded locations. Consideration of environmental characteristics is critical for planning improved measures for Leishmaniasis control. The importance of environmental characteristics in transmission foci has been considered in several studies (Teodoro et al. 2004). Environmental characterisation and management strategies need to be incorporated in any VL control programs (WHO 2010)

The 2,427 specimens collected in the Venda Nova SD were allocated to only eight species, and of these, 95.3% were *Lu. longipalpis*. This species was collected in 15 of the 16 coverage areas sampled in the urban area and was the most abundant in all 15. These findings support the suggestion that urbanization be regarded as a risk factor for the occurrence of the transmission cycle of *L. infantum* in areas of the Brazilian Cerrado, particularly when natural processes are disrupted by anthropogenic disturbance (Cattand et al. 2006).

Although in low abundance, suspected or proven vectors of *L. braziliensis* were among the species collected at Venda Nova SD. *Ny. whitmani*, an important vector of *L. braziliensis* (Rangel & Lainson 2009), was recorded and represented 0.37% of the total sample collected in Venda Nova SD. The possibility of *Ny. whitmani* participating in the transmission cycles of *L. infantum* needs to be clarified (Margonari et al. 2010, Saraiva et al. 2010).

Specimens of *Ev. sallesi* and *Ev. cortelezzii*, as well as the cortelezzii complex, were also recorded in low abundance in Venda Nova SD, totaling less than 3% of all the phlebotomines collected. Species of the cortelezzii complex are suspected of participation in the transmission of *L. braziliensis* and *L. infantum* to humans (Nascimento et al. 2013).

*Ev. lenti* had a low abundance with only two specimens being collected. This species is frequently collected in areas of *Leishmania* transmission and *L. braziliensis* DNA was detected in this species in a study conducted in the state of MG (Margonari et al. 2010). This species can be used as an index species for the occurrence of *Lu*. *longipalpis* (Pinto et al. 2012).

Only one specimen each of *Sciopemyia Sordellii* (Shannon & Del Ponte, 1927), *Micropygomyia scheberi* (Martins, Falcão & Silva, 1975) and *Lu. carvernicola* (Costa Lima, 1932) were collected. These records are surprising because these species are not known to exhibit synanthropic behavior and are not commonly reported in urban areas (Pinheiro et al. 2013). One possible explanation is trap contamination, but this is highly unlikely because the traps used were used only in Venda Nova SD. *Lu. cavernicola* and *Sc. sordelli* are associated with cave environments, but the former species has also been recorded in other ecotypes such as forests and pastures (Carvalho et al. 2010, Rego et al. 2014). There is no evidence that these species participates in the transmission cycles of *Leishmania* sp. Likewise, there is no evidence that *Mi. schreiberi* participates in the transmission cycle of leishmanniasis. The single specimen of *Mi. schreiberi* collected in Venda Nova SD was recorded in a cattle shed (VNSVCU), which is consistent with other findings about this species (Souza et al. 2009).

The ecological characteristics of the sand fly fauna of Venda Nova SD, low species richness, diversity, evenness and the expressive dominance of *Lu. longipalpis*. show how anthropogenic influences may result in increased exposure of human populations to sand fly vectors and, as a consequence, an increased risk of being infected by *Leishmania* (Cattand et al. 2006). The species accumulation curve of Venda Nova SD stabilised late (at the 19th sampling event) and with low species richness even though there was a great sampling effort. This urban area also had a considerably low level of evenness, which helps to explain these characteristics of the species accumulation curve.

Epidemiological data for Venda Nova SD, and the pattern of *Lu. longipalpis* occurrence reinforces the statement that this species is the main vector of *L. infantum* in Brazil. Since the 1960’s several studies have indicated morphological differences among populations of this species and recently there have been studies that indicate that *Lu. longipalpis* is a complex of species varying in morphological, genetic and behavioral characteristics (Mangabeira & Herlock 1961, Bauzer et al. 2002). In previous studies of sand flies in BH, *Lu. longipalpis* was found to have anthropophilic tendencies, with high abundances and high rates of natural infection. The data presented herein corroborate other studies that have demonstrated that *Lu. longipalpis* has a great ability to adapt to an anthropogenically modified environment and that it tends to be the most abundant species in the urban areas where it occurs (Oliveira et al. 2003, de Souza et al. 2004).

*Lu. longipalpis* was the dominant species in every area where sand flies were collected and in every month of the study. The species has been recorded both in locations with a high number of environmental characteristics favorable to the occurrence of phlebotomines, and in locations that did not have many of these characteristics.

The pattern of the seasonal curve of sand flies in Venda Nova SD is strongly determined by *Lu. longipalpis*. The peaks in phlebotomine collection match the warmer and wetter periods. Absence of statistically significant correlation between the seasonal curve and climatic factors may indicate the need for more a detailed evaluation of the climatic parameters. Studies indicate that the use of weekly evaluations and the local collection of climate data are preferred (Rego et al. 2014). Unfortunately, local climate data were not collected in the present study and weekly data were not available. Interestingly, the pattern of seasonal variation in phlebotomines in this study differs from other studies conducted in BH (de Souza et al. 2004). These data demonstrate that the surveillance and control of the leishmaniasis needs to have constant, or periodic, sand fly population monitoring.

The natural infection rates of *Lu. longipalpis* by *L. infatum* and *L. braziliensis* agree with the epidemiological scenario of serological positivity in dogs of the SD (Margonari et al. 2006, PBH 2014). The possibility of *Lu. longipalpis* acting as a vector of *L. braziliensis* requires further study. *Lu. longipalpis* is permissive to the development of various *Leishmania* species in laboratory (Walters et al. 1993).

The interpretation of the data presented in Table IV makes clear the difficulty of obtaining satisfactory identification of leishmanial DNA from material extracted from phlebotomine. Even with the low quality of the data, they were presented as being consistent with the epidemiological situation of the SD.

Currently, geoprocessing tools have been used extensively in studies of disease occurrence and associated factors (Margonari et al. 2006, Araújo et al. 2013). In a study conducted throughout the city of BH from 2001 to 2002, spatial correlation was found among the greater abundance of sand flies, the greater occurrence of human cases of LVH, and the greater occurrence of *Leishmania* seropositive dogs (Margonari et al. 2006). The descriptive vector density map and the analysis of *Leshmania* DNA detection indicate that VNSM, VNJE, VNSA and VNMC are the areas with the highest risks when considering the variables related to vectors.

The numerous previous studies on the sand fly fauna of the city of BH have had different sampling strategies, were performed in different locations, and had different results (de Souza et al. 2004, Resende et al. 2006, Saraiva et al. 2011). The present work is the most detailed survey of the sand fly fauna of a SD to date, and it demonstrates the amount of variation that exists in species richness, species abundance, and natural infection rates among the sampled areas and among the studied time periods. Furthermore, it demonstrates the necessity of introducing a systematic monitoring of sand flies in order to evaluate LV control strategies, particularly concerning the chemical control of its vectors.
